# Electrodeposited Hybrid Biocathode-Based CO_2_ Reduction via Microbial Electro-Catalysis to Biofuels

**DOI:** 10.3390/membranes11030223

**Published:** 2021-03-22

**Authors:** Abdul Hakeem Anwer, Nishat Khan, Mohammad Faisal Umar, Mohd Rafatullah, Mohammad Zain Khan

**Affiliations:** 1 Industrial Chemistry Research Laboratory, Department of Chemistry, Faculty of Sciences, Aligarh Muslim University, Aligarh 202002, India; hakeemanwer1@gmail.com (A.H.A.); nishatk57@gmail.com (N.K.); 2 School of Industrial Technology, Universiti Sains Malaysia, Penang 11800, Malaysia; faisalumar@student.usm.my

**Keywords:** CO_2_ reduction, bioelectrochemical cell, reductive catalytic current, green biotechnology, acetic acid

## Abstract

Microbial electrosynthesis is a new approach to converting C1 carbon (CO_2_) to more complex carbon-based products. In the present study, CO_2_, a potential greenhouse gas, was used as a sole carbon source and reduced to value-added chemicals (acetate, ethanol) with the help of bioelectrochemical reduction in microbial electrosynthesis systems (MES). The performance of MES was studied with varying electrode materials (carbon felt, stainless steel, and cobalt electrodeposited carbon felt). The MES performance was assessed in terms of acetic acid and ethanol production with the help of gas chromatography (GC). The electrochemical characterization of the system was analyzed with chronoamperometry and cyclic voltammetry. The study revealed that the MES operated with hybrid cobalt electrodeposited carbon felt electrode yielded the highest acetic acid (4.4 g/L) concentration followed by carbon felt/stainless steel (3.7 g/L), plain carbon felt (2.2 g/L), and stainless steel (1.87 g/L). The alcohol concentration was also observed to be highest for the hybrid electrode (carbon felt/stainless steel/cobalt oxide is 0.352 g/L) as compared to the bare electrodes (carbon felt is 0.22 g/L) tested, which was found to be in correspondence with the pH changes in the system. Electrochemical analysis revealed improved electrotrophy in the hybrid electrode, as confirmed by the increased redox current for the hybrid electrode as compared to plain electrodes. Cyclic voltammetry analysis also confirmed the role of the biocatalyst developed on the electrode in CO_2_ sequestration.

## 1. **Introduction**

Continuously increasing amounts of CO_2_ emission is a major global issue that must be addressed soon [1,2]. CO_2_ (~60%), CH_4_ (~22%), and N_2_O (~3%), along with other trace gases, contribute to global warming, causing an elevation in the Earth’s surface temperature [3]. Researchers are developing several strategies to mitigate high CO_2_ levels such as low-carbon renewable energy source development, minimizing the usage of fossil fuel, electro-catalysis, chemical scrubbing, carbon capture and utilization (CCU), or carbon capture and storage (CCS) [4]. Microbial electrosynthesis systems (MES) are the latest attraction for CO_2_ sequestration. MES is a bioelectrochemical system (BES) that acts as a perfect carbon fixing unit reducing simple C-1 gases such as CO_2_ and CO to more complex multi-carbon compound such as alcohols (ethanol), volatile fatty acids (acetic acid), and solvents with the aid of microbial catalyst [5,6,7]. The attractiveness of MES lies in its ability to fix CO_2_ and simultaneously produce value-added products. MES is a double-chambered system consisting of anode and cathode compartments, where acetogenic microbes at the cathode catalyze the reduction of CO_2_ via using electrons and protons generated from cathode and electrolyte, respectively. The most important part is played by the microbial catalyst in MES as it fixes CO_2_ to multi-carbon products via the Wood–Ljungdahl (WL) pathway [7,8,9,10]. MES, not being thermodynamically feasible, requires a minimum energy to overcome the thermodynamic barrier in order to derive value-added chemicals from CO_2_ reduction [11,12,13]. Usually, the MES principle follows the enrichment of biocatalyst on the surface of electrode-catalyzing CO_2_ reduction via the bioelectrochemical process through controlled electron flux by controlling either applied potential or current. In MES, several variables such as pH, applied voltage/current, biocatalyst, electrode materials, counter electrode, reactor configuration, etc. may generally regulate the nature of product synthesis [14].

In MES, electrode material and electrotrophy are conjunctively playing a key role as the bioelectrochemical reactions occurring within the system are regulated by the biofilm formed on the electrode surface [15]. Based on the operative factors, any electrode can act as an acceptor or donor of electrons. In MES, the cathode acts as a working electrode and functions as an electron donor. The electrodes, on the other hand, are withdrawn from the anode, which plays the role of counter electrode operating under controlled current /voltage [16]. The surface-bound biofilms catalyze the effective reduction of CO_2_ to synthesize value-added products (VFA) [17]. Capacitance or capacity of holding electrons also depends on biofilm adhesion on the electrode surface [18]. The CO_2_ reduction to form products in MEC is also affected by the capacitance of the electrode. The products formed in MES depend on the applied voltage and electron flux [19,20]. Most MES studies have by far focused on the production of acetate as it is the primary product synthesized [21,22]. In MES, capacitance, electrotrophy, and electron flux are dependent on various electrode properties such as material, composition, porosity, biocompatibility, anti-corrosive, mechanical strength, surface area, impurities, and others that in turn catalyze the bioelectrochemical reactions on their surface [18]. The relation between capacitance and electrotrophy in MES for CO_2_ reduction is still limited.

The biocathode plays a crucial role in electron transfer and overall performance of the MES as the microbes interact directly with the biocathode for electron transfer and CO_2_ reduction. Several attempts have been made to improve the electron exchange between the microbes and electrode. Coating the electrode surface with modifiers such as PANI, PPy, CNT, and chitosan can provide a larger surface area and enhance the biofilm development and electron transfer, thereby enhancing the system efficiency [23,24,25]. Metal catalysts such as Ni, Au, and Pd can improve the electron transfer by lowering the activation energy [26,27]. In the present study, we explored various biocathodes viz. carbon felt (CF), stainless steel mesh (SS), CF and SS merger (CF/SS), and an electrodeposited hybrid electrode (CF/SS/Co-O) composed of CF, SS, and a cobalt oxide (Co-O). The choice of CF was made due to its high surface area and conductivity [28]. Compared to the carbon electrodes, SS has higher conductivity and mechanical strength with being low cost [23]. Further, cobalt with multiple oxidation states can enhance the electron shuttling between electrodes and microbes [29]. The study, for the first time, explores the collective effect of the electrode modifiers on the performance of MES. The study analyzes and discusses the key functioning parameters such as electrochemical impedance, electrotrophy, reductive behavior, and catalytic currents of biocathodes used. Comparative analysis of biochemical and electrochemical parameters for four different biocathodes was also conducted to evaluate the optimum biocathode material and increasing the MES performance.

## 2. Materials and Methods

### 2.1. Chemicals

Analytical grade hydrochloric acid (HCl), sodium bicarbonate (NaHCO_3_) from SRL India, cobalt chloride (CoCl_2_), boric acid (H_3_BO_3_), sodium hypophosphite (Na_2_H_2_PO_2_) and sodium chloride (NaCl) from Merck, Gujarat, India, and deionized water were used during this study.

### 2.2. Electrode Preparations and Morphology, Elemental Characterization

The cobalt oxide (Co-O) modified electrode CF was prepared via a PGSTAT204N, (Metrohm Autolab, Netherlands) potentiostat using electrochemical deposition, as reported in earlier procedures [30,31]. The electrochemical deposition was performed using a three-electrode device consisting of a working electrode as an electrodeposit substrate, Ag/AgCl reference electrode (1 M KCl), and platinum (Pt) as a counter electrode purchased from Metrohm Autolab, Netherlands, Instruments. A cathodic electrochemical deposition on a CF was used to prepare the CF+ Co-O catalyst. The deposition solution contains 0.1 M NaCl, 0.33 Na_2_H_2_PO_2_, 0.2 M CoCl_2_, and 0.15 M H_3_BO_3_. The CF was sequentially washed with the isopropanol and acetone and then immersed in deionized water prior to electrodeposition. Surface morphology was studied by FESEM, Japan (JEOL model JSM-6510) and for elemental analysis by Oxford Instruments INCAxsight EDAX spectrometer [32,33].

### 2.3. Inoculation

A homoacetogenic chemolithoautotrophic cultivated mixed inoculum was obtained from a previous MES setup continuously working in our laboratory for CO_2_ sequestration mode [12]. The unwanted methanogenic microbial population was inactivated from anaerobic sludge via two-stage enrichment of chemolithoautotrophic microbes. Mixed consortia pretreatment was conducted via the addition of 2 mL (from 500 mM stock solution, to inhibit growth of methanogens) 2-bromoethanesulfonic acid (BESA), supplemented with H_2_ and CO_2_ in the second stage. Prior to inoculation in MES, pre-treated culture was revived via inoculation of 100 mL of synthetic wastewater with 2 g/L of sodium bicarbonate as a carbon source [5,34].

### 2.4. MES Configuration and Design

Four similar double-chambered MES reactors were made up of polyacrylic material with a working volume of 200 mL. Nafion 117 (proton exchange membrane) was used to isolate the anode and cathode chambers. For electrode placing, sample collection, and N_2_ flushing, rubber stoppers with two small holes were mounted to each container to provide flexibility. Nitrogen (N_2_) gas (approx. 99.9%) was discharged into the MES of headspace for 10 min before the operation of the system and after collecting the sample to maintain the anaerobic environment of the system [35]. The configuration of electrodes in MES varied with various configurations of functioning electrodes (biocathode) against counter electrodes (anode; carbon felt). Different biocathode (cathode size was 3.50 cm × 1.50 cm) such as carbon felt (MES-1, CF), stainless steel mesh (MES-2, SS), CF/SS prepared by intertwining SS mesh over CF manually (MES-3, CF/SS), and CF/SS/Co-O prepared by electrodeposited cobalt oxide (Co-O) on carbon felt and stainless steel (MES-4, CF/SS/Co-O). Before use, all the cathodes were exposed to acid (0.10 N H_2_SO_4_; for 10 min) to eliminate the unnecessary particles on the surface impurities to increase the electrode activity [8]. To maintain connectivity with the electrodes, titanium wires thickness (1 mm) as a current collector were used and packed with epoxy sealer. Formed a synthetic wastewater: 1.5 g/L of KH_2_PO_4_, 0.5 g/L of NH_4_Cl, 2.9 g/L of K_2_HPO_4_, 25 mg/L of CoCl_2_, 0.3 g/L of MgCl_2_, 11.5 mg/L of ZnCl_2_, 5 mg/L of CaCl_2_, 10.5 mg/L of CuCl_2_, and 15 mg/L of MnCl_2_ [36,37] was used as electrolyte in the four experimental MES setups. The pH and concentration of the phosphate buffer solution were respectively 6.9 and 30 mM. Prior to each cycle, bicarbonate (HCO_3_^−^), a form CO_2_, was added as a single source of carbon at 5 gm/L, which is equivalent to the 3.6 gm/L of CO_2_. In order to preserve the anaerobic environment, L-cysteine and sodium thioglycolate concentrations of 20 mM were added to the electrolyte as dissolved oxygen scavengers. A leakage-proof sealer was placed around the rubber stopper before operation to maintain stringent anaerobic conditions [5,12].

### 2.5. Operation

Four MES were operated in batch mode on the different working electrodes at a constant applied potential of −0.8 V (vs. Ag/AgCl (3.5 M KCl)). Using the potentiostat/galvanostat, cyclic voltammetry and chronoamperometry analyses were performed. Non-turnover or blank electrochemical output in the absence of a biocatalyst was reported for all MES. After completion of the blank experiment, 10% enriched chemolithoautotrophic bacterial inoculum was injected in all MES and run for 10 cycles, with 72 h for each cycle run time at room temperature (28 ± 3 °C). HCl (1N) and NaOH (1N) were used to adjust the pH of the electrolyte to 6.8 ± 0.2 prior to each cycle feeding process. Homogeneity of the inoculum and electrolyte was maintained with the help of a stirrer at 120 rpm. The components of the MES after each cycle were permitted to accumulate, followed by the supernatant removal while the inoculant was reused for the next cycle. The biofilm developed on the electrode surface was not disturbed during the feed replacements. The liquid samples were evaluated to estimate the product concentration according to conventional approaches (APHA, 1998) [5,8].

### 2.6. Analysis

The MES output was assessed periodically (12 h) by evaluating the parameters such as biofuel (acetic acid and ethanol) and pH for the collected samples. The development of biofuel (acetic acid, ethanol) was inspected by using the NUCON 5700 gas chromatograph (GC) connected to a flame ionization detector (FID) and a column Chromosorb101 operated as previously reported by Anwer et al. [12]. Before injecting the sample for analysis, it was filtered by the 0.2 µm syringe, and then 10 µl sample volume was injected for each evaluation. The electrochemical properties of the MES were analyzed by using the chronoamperometry (CA) of MES performed for electrochemical characterization at an applied potential of −0.8 V (vs. Ag/AgCl) on the working electrode.

Cathodic electron efficiency, coulombic efficiency (CE) is an indicator of the efficacy of electron capture by microorganisms from the electric current to form materials and was estimated using Equation (1) [5,38].

(1) $$\mathrm{CE}=\frac{n_{pro}\times f_{pro\;\times \;}F}{\int_{t_{o}}^{t}Idt}\times 100$$

where CE is the coulombic efficiency (in %), $f_{pro}$ is the molar conversion factor (8 acetate electron equivalent), F is the constant of Faraday (96,485.3 C mol^−1^), and I is the current supplied to the poised cathode.

Carbon recovery efficiency (CRE, $\;\eta_{c}$) or carbon fixing represents the percentage of carbon used by microbes to synthesize organic products from biogas. The efficacy of carbon recovery was measured using Equation (2) [39].

(2) $$\eta_{c}=\frac{n_{pro}\times f_{c,pro\;}}{n_{gas}}\times 100$$

where *η_c_* is the performance of carbon recovery (%), $f_{c,pro\;}$is the amount of moles of carbon in a commodity mole (for example, 2 moles of carbon in one acetate mole), and $n_{gas}$ is the CO_2_ moles in the gas.

The reduction in bicarbonate amount was monitored according to the standard methods (APHA, 1998) [40]. However, the generation of reductive catalytic currents relative to applied voltage was analyzed using the technique of chronoamperometry (CA), and the phenomena of electron discharge were investigated by cyclic voltammograms (CV) at a 10 mV/s of scanning rate [38]. The specific capacitance of the cathode was evaluated using the following Equation (3) [41].

(3) $$Specific\;capacitance\;\left(C\right)=\frac{I_{charge-discharge\;}\times t}{U_{charge-discharge}\times A}$$

## 3. Results and Discussion

### 3.1. Characterization of Modified Carbon Felt

**Figure 1:**
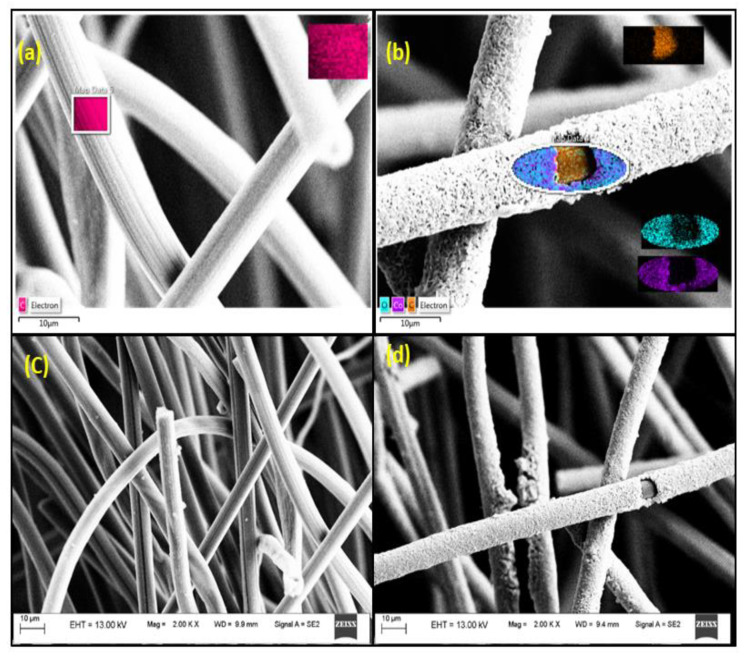
Energy-Dispersive X-Ray Spectroscopy **(**EDS) image of (**a**) bare carbon felt, (**b**) Co-O electroplated on carbon felt (CF)**,** elemental mapping images show in inset—carbon (C), cobalt (Co), and oxygen (O). (**c**) SEM image of bare CF and (**d**) cobalt oxide modified CF.

CF/SS/Co-O for MES-4 nanoparticles were fabricated as described earlier onto the CF electrode [30]. As shown in Figure 1b, the nanocomposite Co-O was collected on the CF after electroplating. Elemental mapping analysis images of the electrospinning film are shown in Figure 1, depicting carbon (C), cobalt (Co), and oxygen (O). Cobalt and oxygen were deposited uniformly on the surface of carbon felt in orange and light blue color, respectively. Compared with Figure 1c, it is clear in Figure 1d that cobalt oxide was deposited on the surface of the CF, which is in arrangement with the SEM image. The bare CF anode image (Figure 1c) displays the comparatively smooth surface of cross-linked carbon fibers. The CF/Co-O cathode image (Figure 1d) indicates the formation of a well-mannered Co-O layer to the CF electrode after electrodeposition. On the CF/Co-O cathode (Figure 1c), the Co-O sheets were densely warped on the surface of CF fibers to form a thin crumpled structure, which substantially increased the surface area of the cathode. Figure 1d shows the image of the CF/Co-O cathode, with the formation of Co-O particles being observed on CF surfaces.

### 3.2. Responsive Reductive Current Generation

**Figure 2:**
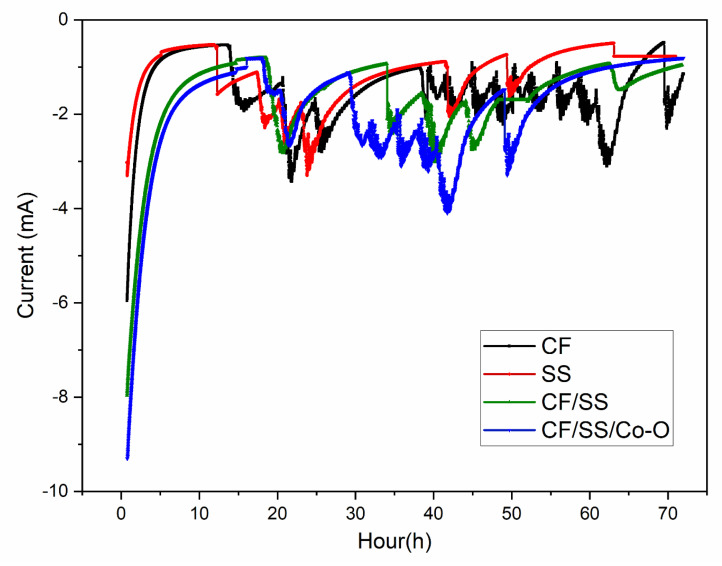
Evaluation of bioelectrochemical catalytic currents through chronoamperometric current profiles of different microbial electrosynthesis systems (MES) operated with varied biocathodes.

In the process of bioelectrochemical conversion of CO_2_ to produce value-added products (acetic acid and ethanol), it was observed that the generation of reductive catalytic current is affected by the type of biocathode used. As shown in Figure 2, the electrodeposited hybrid electrodes were observed to display a higher magnitude of reductive current of −9.2 mA for CF/SS/CoO and −7.8 mA for CF/SS as compared to −5.9 mA for CF and −3.2 mA for SS, which well depicts the microbial electrosynthesis reaction as the CO_2_ reduction proceeds [42]. Further, CA analysis also illustrates the nature of the eletrotrophy or the flow of electrons (current production) over a period of time. It was observed during the study that the CF/SS/Co-O biocathode operated for an extended period of time with a continuous increase in reductive catalytic current, which is in agreement with increased product yield (acetic acid, ethanol, etc.). The higher current output and product yield could be a result of the enhanced porosity and pliable nature of the electrodeposited hybrid electrode [40,43]. As all the MES were operated with the same biocatalysts, the variation in all the system performances can be directly linked with the various biocathodes explored during the study [44]. It was observed that the reductive catalytic current for CF/SS/Co-O was high over time, while for CF/SS, the initial current of −8 mA stabilized over time with a slight decrease (−3 mA) and again stabilized at −1 mA for the rest of the experimental operation. On the other hand, the CF biocathode showed a decrease in the reductive current to −3.5 mA from the initial current value of −5.7 mA, which further declined to finally stabilize at −2 mA for the rest of the experiment, while for the SS electrode, the current values were quite low from the beginning (−3.2 mA), which decreased to -0.85 mA and then decreased to −0.55 mA for the rest of the experiment. This result indicates that the bioelectrochemical reduction was conducted by the biocatalytic operation of the cathode electrode-attached microorganisms. The electrodeposited hybrid electrode (MES-4) generated a high reductive current as compared to other MES, which directly correlated the production of acetic acid. In relation to the acetic acid synthesis rate achieved in bare CF (MEC-1) compare to MES-4 during the same time, this indicated a 50.45% improvement.

### 3.3. Acetic Acid

**Figure 3:**
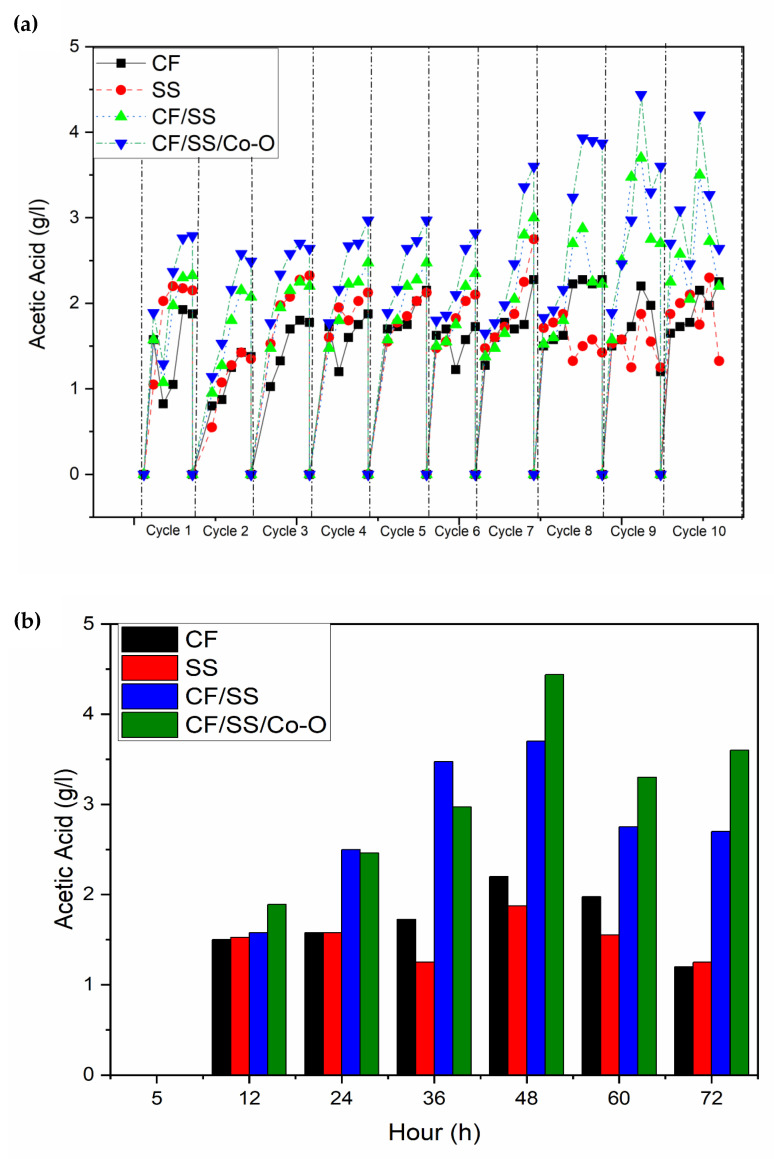
Acetic acid profile. (**a**) MES operated with different biocathodes for ten consecutive cycles. (**b**) A batch cycle with an HRT of 72 h.

Four MES setups were assembled and operated with varying biocathodes. The systems were inoculated with selectively treated and acid-treated microbial inoculum. The MES reactors were operated at the applied potential of −0.8 V Vs Ag/AgCl (s) against the working electrode (biocathode) through the potentiostat-galvanostat system, while the anode acted as the counter electrode. The performance of MES with different biocathodes was analyzed in terms of acetic acid production. The bioelectrochemical reduction of CO_2_ to acetic acid, as quantified with the help of GC, depicted that the acetic acid production varied with the biocathode used, suggesting the role of the biocathode in the potential performance of MES. Through GC analysis, the CF/SS/Co-O biocathode was observed to yield 4.4 g/L of product titre, which was observed to be highest as compared to other biocathodes tested, such as CF/SS (3.70 g/L), SS (1.87 g/L), and CF (2.2 g/L). The generation of acetic acid observed for a single batch cycle is presented in Figure 3b, while the production rate for the most efficient cycles is presented in Figure 3b. From Figure 3b, it can be observed that the acetic acid generation increased with increasing cycle length, peaked at 48 h, and started to decrease thereafter before becoming constant. The decrease in the acetic acid concentration beyond 48 h can be attributed to its subsequent conversion to other value-added chemicals such as ethanol and other simple organic acids. Further, the alteration in acetic acid production with varying biocathodes could be attributed to the difference in the enrichment of biofilm developed on the surface, which in turn affects the electrotrophy enabling the surface-active bioelectrochemical process. The performance of biocathodes CF/SS/Co-O and CF/SS were observed to be similar in terms of acetic acid production; however, the electrotrophy and catalytic current generation were observed to be higher for CF/SS/Co-O as compared to CF/SS, which affected the bioelectrocatalytic reduction of CO_2_. Alternatively, electrolysis on the surface of the electrode might be responsible for the lower acetic acid yield observed with the SS electrode, which could be confirmed with the increase in pH as a result of the rise in OH^-^ ions [45,46]. These electrotrophs, in turn, might aid in bioelectrochemical CO_2_ reduction to produce acetic acid at the modified electrode as compared to the plain electrode. Biofilm formation started from cycle 4 and continued until cycle 10 in MES-1, MES-3, and MES-4, although in the case of MES-2, loosely bound biofilm was observed until the end of the operation. Maximum overall acetic acid productivity of 2.2 g/L (at 48 h) was observed in MES-1 during the eighth and ninth cycle, and a decrease in biosynthesis was observed in extended retention time activities, such as 1.25 g/L at 72 h. In MES-2, biosynthesis of 1.87 g/L at 48 h and 1.21 g/L at 72 h was observed, same as for MES-3 setup 3.7g/L at 48 h and 2.7 g/L at 72 h. The cobalt modified electrode-equipped reactor (MES-4) displayed productivity of 4.40 g/L acetic acid at 48 h and followed a similar pattern in further extended operation, i.e., 3.6 g/L at 72 h. Among all the MES, maximum total acetic acid production was found in MES-4 (4.40 g/L) followed by MES-3 (3.7 g/L), then MES-1 (2.2 g/L) and MES-2 (1.87 g/L).

Various studies have reported earlier the performance of MES with various electrodes such as CF (9.8 g/L/d) [47], CF/SS (1.3g/L/d) [39], CF (0.06g/L/d) [17], graphite (2.1 g/L) [8], and graphite granules (1.04 g/L/d) [48] etc., shown in Table 1, attaining substantial production rate of acetate. The performance of the CF/SS/Co-O electrodeposited hybrid electrode has not been evaluated before. The batch mode MES performance relatively improved with the hybrid electrode with comparatively good acetic acid production. The addition of Co-O to the CF/Co-O electrode significantly improved the acetate production rate as compared to the plain CF electrode. The improvement in performance could be attributed to the functionalization of Co-O as supporting substratum promoting biofilm formation and eventually electrotrophy. Further, the incorporation of Co with CF improved capacitance and microbial electrosynthesis.

### 3.4. Ethanol

**Figure 4:**
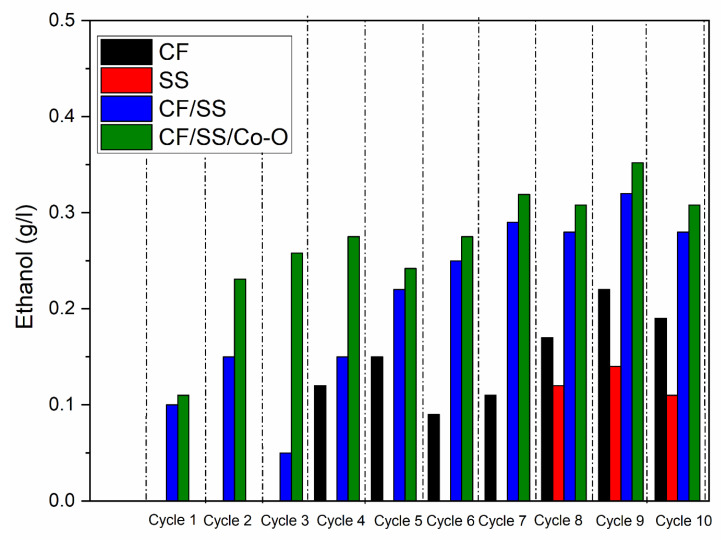
Ethanol in MES operated with varied biocathodes.

In addition to the acetic acid generation in MES operated with the CF/SS/Co-O hybrid electrode, synthesis of ethanol was also observed in a small amount with the yield of 0. A total of 352 g/L for MES-4, MES-3 (0.32 g/L), and MES-2 (0.14 g/L) as compared to MES-1 (0.22 g/L) in the ninth cycle are shown in Figure 4. However, as compared to other cycles, it produces maximum ethanol same as acetic acid produced in the ninth cycle. The presence of ethanol could be from the synthesized acetic acid reduction [54]. The pH profile recorded was also in agreement as the acetic acid accumulation prompted the synthesis of alcohol. It has already been reported in previous studies that lower pH favors the reduction of acetic acid to produce ethanol [55]. The production of ethanol and acetic acid both in MES with CF-based electrodes might be attributed to the similar microbial community developed on the electrode surface constituting acetic acid and ethanol producers. Table 2 presents the electrochemical and biochemical performances of MES with varying electrodes.

### 3.5. Redox Profile

**Figure 5:**
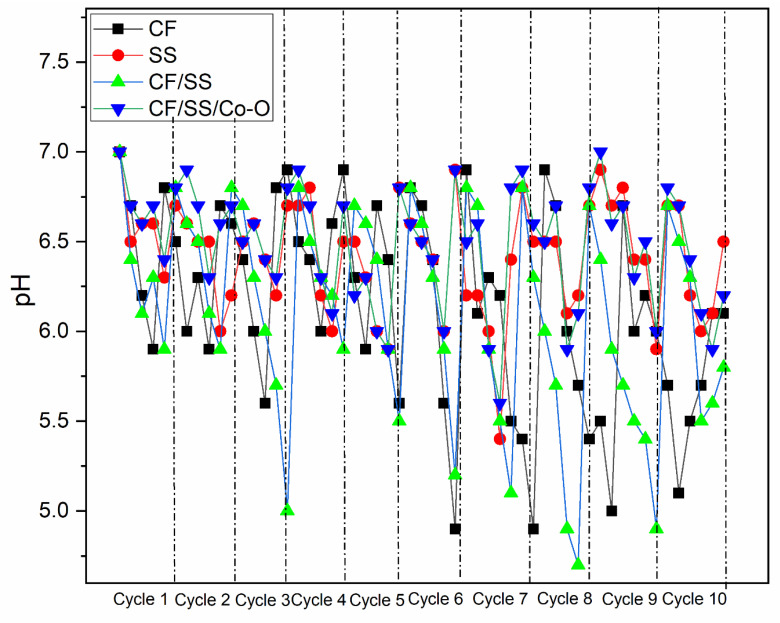
Change in redox activity interpreted as pH for 10 consecutive MES cycles operated with various biocathodes.

The transformation of CO_2_ to value-added chemicals is significantly affected by the redox environment that affects acetic acid production by directing the metabolic activities of the microbes involved. The pH trend varying with cycle number was recorded for the four experimental MES systems and reported in Figure 5. The pH profile, contrary to the profile of VFA synthesis, was noticed to fall as the cycles proceeded for all MES, which could be directly linked to the presence of synthesized acetate [56]. For all MES, the initial pH was adjusted to 6.8 ± 0.2 to create a favorable redox environment to support the metabolic activities of microbes [57]. The decreasing trend of pH for MES was recorded with MES-4 reaching a value of 5.0 ± 0.2 till the eighth and ninth cycle, whereas 5.15 ± 0.1, 5.5 ± 0.1, and 6.11 ± 0.1 for MES-3, MES-1, and MES-2, respectively. However, the decrement was balanced due to HCO_3_^−^ion reversible-binding capacity with H^+^ ions in solution, effectively buffering to support acetic acid production [6]. The pathway of acidogenesis toward solventogenesis was also drifted by a higher concentration of proton abundance at a low pH and the constant availability of electron flux by under-controlled potential [40]. Thereafter, an increase in pH in all MES is found, which may be due to the usage of acetic acids by other bacterial species as a carbon source.

### 3.6. Carbon Conversion Efficiency

The rate of substrate conversion is one of the key factors deciding the product formulation performance of MES [58]. The ratio of product carbon equivalents to the provided carbon represents the biological CO_2_ conversion efficiency to acetic acid [59]. The decrement in the concentration of substrate was observed to vary with the different electrodes tested. MES-4 reported the highest substrate conversion and product formation rate. The conversion of CO_2_ to acetate followed the trend: MES-4 (75.9 ± 0.5%) > MES-3 (69 ± 0.5%) > MES-1 (56 ± 0.5%) > MES-2 (39 ± 0.5%). A similar conversion trend was also observed in terms of ethanol with the conversion efficiency of 13.2 ± 0.1% for MES-4 followed by MES-3 (12.1 ± 0.1%), MES-1 (6 ± 0.1%), and MES-2 (5 ± 0.3%), respectively (Table 2). Further, the cumulative carbon conversion efficiency could be ascribed to the enhanced biocatalytic activity on the electrode surface as the biofilm development was more favored at the electrodeposited hybrid electrode. In MES, acetic acid production directly correlates to diminishing substrate, indicating that the fraction of carbon reduced contributed to the formation of products. The equation below presents the stoichiometric conversion of CO_2_ to VFA conversion in MES [6,60].

2CO_2_ + 8H^+^ + 8e^−^ → CH_3_COO^−^ + H_2_O (4)

2H^+^ + 2e^−^ → H_2_ (5)

C_2_H_3_O_2_^−^+ 5H^+^ + 4e^−^ → C_2_H_5_OH + H_2_O (6)

### 3.7. Bioelectrochemical (BEC) Behavior of MES

**Figure 6:**
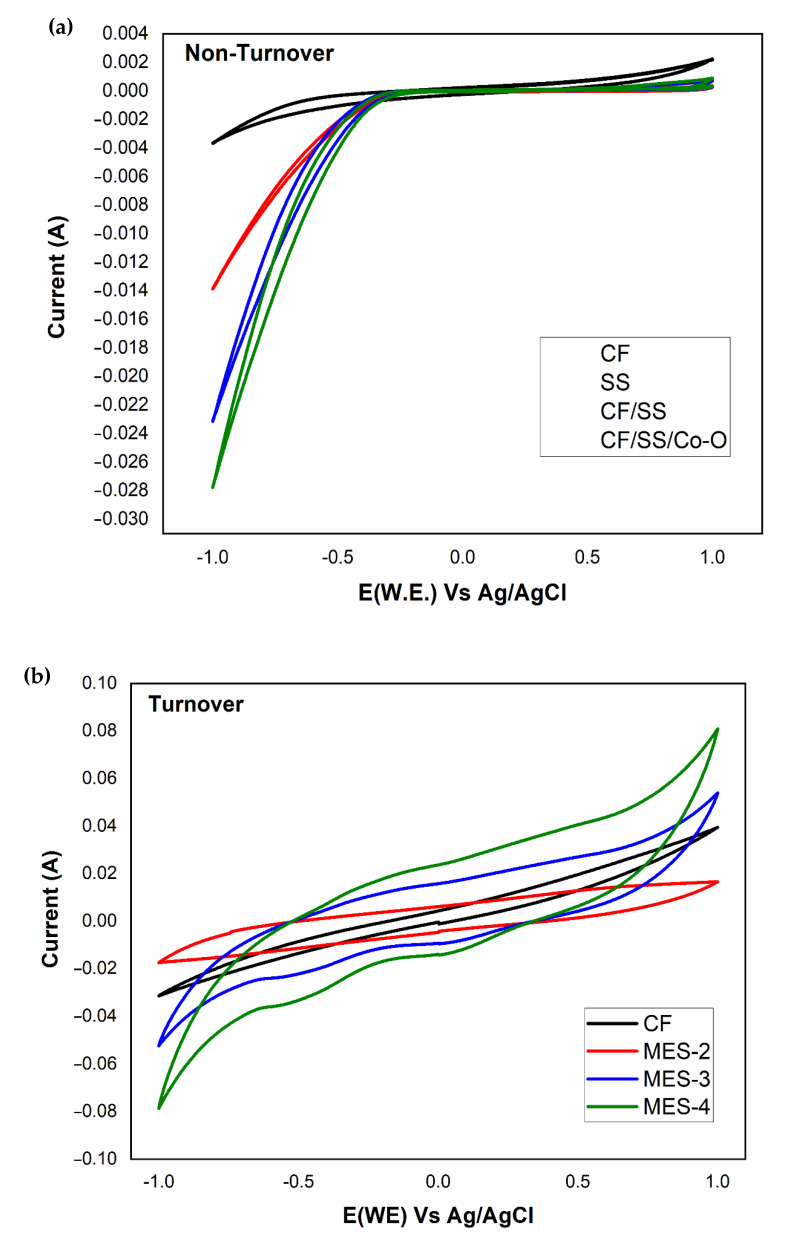
Cyclic voltammograms; (**a**) non-turnover and (**b**) turnover for the different electrode materials in MES.

In the present work, the BEC response of MES together with variable electrode sequences was analyzed via potential-dynamic electrochemical techniques such as CA and CV. Non-turnover CVs were recorded for the MES over a potential window of −1 V to 1 V and a rate of 10 mV/s. A 0.002 A oxidative catalytic current was portrayed by non-turnover voltammograms of MES while MES-1 showed a −0.003 A reductive catalytic current (RCC) and MES-2 depicted RCC of −0.013 A and 0.0007 A OCC. In the case of MES-3, an RCC of −0.023 A and OCC of 0.0008 A were recorded. For the MES-4 reactor, −0.028 A RCC and 0.00096 A OCC observing the reductive and oxidative catalytic currents yielded in non-turnover CV led to the deduction that non-faradic reactions were occurring inside the electrolyte (Figure 6a). After the non-turnover CVs were taken into account, the MES were injected with inoculum (biocatalyst), and the turnover CV was measured over a steadied performance toward reductive current generation. It was seen that the turnover CV of all MES displayed elevation in both the OCC and RCC in comparison to the reference (non-turnover). These outcomes advocated that the biofilm creation over the working electrode and association of electrode-microbe interactions led to the biological sequestration of CO_2_ in charge transfer kinetics. Higher OCC (0.079 A) and RCC (−0.077 A) were observed in MES-4 followed by MES-3 (OCC, 0.053 A; RCC, −0.0517 A), MES-1 (OCC, 0.031 A; RCC, −0.037 A), and MES-2 (OCC, 0.016 A; RCC, −0.017 A) (Figure 6b) when the turnover voltammograms of four MES were compared. These redox currents in the MES demonstrate the charge transfer productivity of biocatalyst biocathode kinetics against substrate reduction. Acetic acid production rates correlated with the redox currents that were generated in all four MES. The greater value of redox currents in MES-4 (0.079 A; −0.077 A) represents the improved electrotrophy of the electrodeposited hybrid biocathode, having increased product-formation and substrate-reducing abilities influencing the poised potential simultaneously [5].

### 3.8. Energy Storage in MES

**Figure 7:**
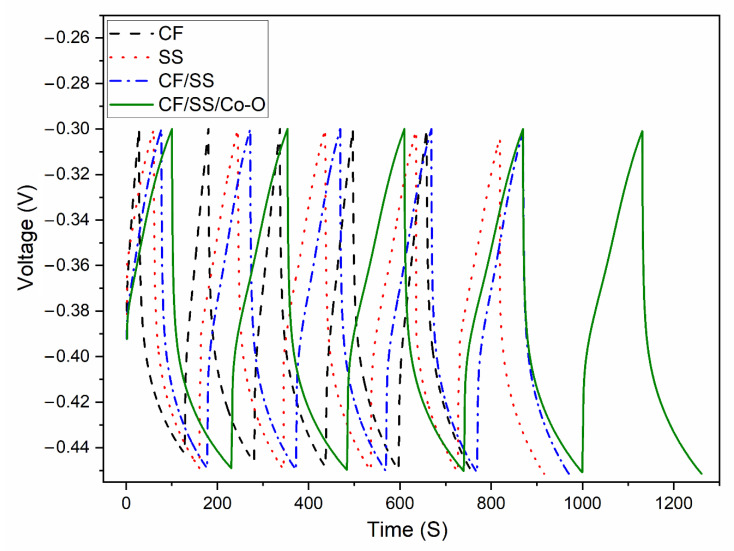
Galvanostatic charge-discharge curves of the MES for all the varied electrode materials.

Galvanostatic charge-discharge (GCD) analyses were carried out to measure the efficiency, durability, and stability of the MES system as a capacitive storage device, and from these tests, the capacitance of the MES was determined by Equation (3). The GCD tests were performed for 5 cycles at 1 mA current density, and the cycle results are shown in Figure 7. A triangular charge-discharge behavior observed in electrochemical supercapacitors describes the capacitive behavior of MES. A pseudocapacitive behavior is the result of electrochemical reversible redox reactions, which may be correlated with a significant deviation of the curve from linearity. Figure 7 shows that the specific capacitance changes from 0.131 F/cm^2^ (MES-2) < 0.147 F/cm^2^ (MES-1) < 0.157 F/cm^2^ (MES-3) < 0.215 F/cm^2^ (MES-4), which may also suggest a gradual increase of specific capacitance (charge storage). An increase in capacitance with modification of the electrode is reported in the earlier publication on supercapacitor electrodes [12,61], and it is also related to ion mass transport limitations, i.e., the change in GCD curve time for high conductive material is the charging potential limit that is reached until electrolyte ions are able to compensate for the charge inside the material’s small pores and less of the electrode surface region is used for charging storage [62]. The specific capacitance of different MES electrodes increases as follows: (MES-2) < (MES-1) < (MES-3) < (MES-4). This indicates a significant change with an increase in specific capacitance with electrodeposited modified CF electrodes. Similar research was performed with different loadings of material by Khilari et al. [63]. This signifies that Co-O plays an active role in electrochemical charge storage on the electrode surface. Increased capacitance is thus due to enhanced electrochemical properties of the Co-O network.

### 3.9. Electrochemical Impedance Spectroscopy (EIS)

**Figure 8:**
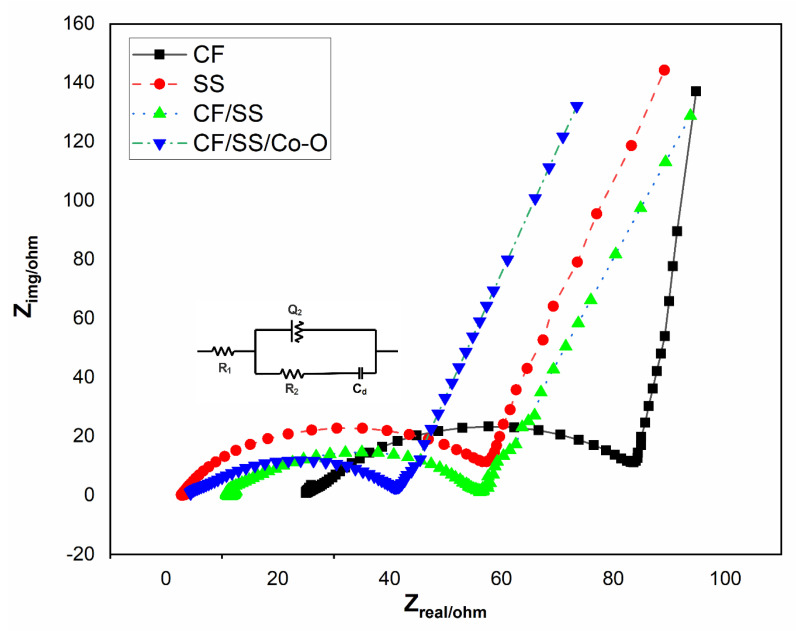
Nyquist plot for MES for all the varied electrode materials. (Rohm: ohmic resistance, R_ct_: charge transfer resistance, Q_2_: constant phase angle element, and C_d_: interfacial capacitance).

The ability of electron transfer can be easily predicted by the EIS spectrum. The faradic and non-faradic components define the overall performance of MES, which includes series resistance (R_s_) comprising of polarization and ohmic resistance and charge transfer resistance (R_ct_), which defines the performance at electrode/electrolyte interface [64]. The Nyquist plot and equivalent Randles circuit for all bare and modified electrodes have been shown in Figure 8 along with EIS parameters for the electrodes under investigation, as shown in Table 3. The R_s_ values for CF, SS, CF/SS, and CF/SS/Co-O were observed to be 25.4, 2.78, 11.1, and 3.92, respectively. The R_s_ was observed to decrease for modified electrodes as compared to bare CF and SS electrodes used. The finding implies that the modification of electrodes improved the EET as compared to the CF and SS electrodes [65,66]. On the other hand, the R_ct_ values were observed to be 65.7, 63.2, 45.9, and 40.2 Ω, respectively, for MES-1, MES-2, MES-3, and MES-4 setup electrodes. The surface modification can be suggested to have decreased the resistances for modified electrodes, which in turn improved the substrate reduction rates at modified electrodes as compared to bare [67]. The lower R_ct_ and thus good charge propagation properties for CF/SS/Co-O could also be attributed to its porous nature [68]. Higher electron conductivity could also be a possible cause of reduced impedance in modified electrodes. Charge transfer resistance is inversely proportional to the standard heterogeneous electron transfer constant, which may have improved for modified electrodes [69]. The modified electrode more easily accepts electrons, thus improving charge transfer and ultimately MEC performance [66]. The result depicts CF/SS/Co-O (MES-4) as the favorable cathode material for catalytic biochemical reduction.

## 4. Conclusions

This research has shown the efficiency of electrodeposited hybrid electrodes (CF/SS/Co-O) as potential capacitive biocathodes that are electrotrophic and will help with better synthesis of acetic acid and ethanol. The significance of electrode materials that allow the formation of electrotrophic biofilm for augmented and controlled transfer of electrons for the synthesis of products (acetic acid and ethanol) was recognized. Electrodeposited hybrid biocathode (MES-4: CF/SS/Co-O) demonstrated a comparatively greater tendency toward reduction capabilities and lower electronic losses than other electrodes (MES-3 (CF/SS), MES-1 (CF), and MES-2 (SS)) toward improved acetic acid synthesis.

## Figures and Tables

**Table 1:** Comparison of results with other microbial electrosynthesis investigations.

Cathode Material	Applied Potential (V vs. SHE)	Biocatalyst	Acetate Production Rate (g m^−2^ day^−1^)	Coulombic Efficiency (%)	Reference
Gas diffusion activated carbon	−1.00	Enriched anaerobic sludge	36.6	35.46	[49]
MWCNT-RVC	−1.10	WWTP sludge	1330	84 ± 2	[50]
Nanoweb 3D RVC	−0.85	WWTP sludge	195 ± 30	70 ± 11	[51]
Carbon felt	−0.90	WWTP sludge	9.75	89.5	[52]
Activated carbon VITO-CoRE^™d^	−0.40	Mix culture	9.49	29.9	[53]
Carbon felt (CF)	−0.8 V	Enriched anaerobic sludge	339.16	40 ± 0.6	Present study
Stainless steel (SS)	−0.8 V	Enriched anaerobic sludge	300.8	36 ± 0.9	Present study
Carbon felt/stainless steel (CF/SS)	−0.8 V	Enriched anaerobic sludge	556.6	52 ± 0.2	Present study
Carbon felt/stainless steel/cobalt oxide (CF/SS/Co-O)	−0.8 V	Enriched anaerobic sludge	622.5	60 ± 0.2	Present study

WWTP—wastewater treatment plant, MWCNTs—multi-walled carbon nanotubes, RVC—reticulated vitreous carbon.

**Table 2:** Comparative efficiency of four different based biocathodes MES configured.

Parameters	MES-1	MES-2	MES-3	MES-3
Working electrode (cathode)	Carbon felt (CC)	Stainless steel mesh (SS)	Hybrid (CF/SS)	Electrodeposited hybrid (CF/SS/Co-O)
Counter electrode (anode)	Carbon felt	Carbon felt	Carbon felt	Carbon felt
Reductive catalytic current (mA)	−5.9	−3.2	−7.8	−9.2
Acetate (g/L)	2.2 ± 0.2	1.8 ± 0.4	3.7 ± 0.4	4.4 ± 0.4
Ethanol (g/L)	0.2 ± 0.02	0.14 ± 0.02	0.32 ± 0.02	0.35 ± 0.02
CO_2_ to acetate conversion (%)	40 ± 0.6	36 ± 0.9	52 ± 0.2	60 ± 0.2
Cumulative carbon conversion (%)	56 ± 0.5	39 ± 0.5	69 ± 0.5	75.9 ± 0.5
Specific capacitance (F/cm^2^)	0.147	0.131	0.157	0.215

**Table 3:** Different resistance components obtained from the Nyquist plot for different biocathodes.

Modified Cathode MES	Solution Resistance (R_s_ Ω)	Charge Transfer Resistance (R_ct,_ Ω)
CF (MES-1)	25.4	65.7
SS (MES-2)	2.78	63.2
CF/SS (MES-3)	11.1	45.9
CF/SS/Co-O (MES-4)	3.92	40.2
